# Supplementary material for: Effectiveness of the pelvic floor muscle training on muscular dysfunction and pregnancy specific urinary incontinence in pregnant women with gestational diabetes mellitus: A systematic review protocol
Source: PLoS One. 2020 Dec 7;15(12):e0241962. doi: 10.1371/journal.pone.0241962 (PMC7721159; doi:10.1371/journal.pone.0241962)
Supplement: S2 File — (PDF) [file pone.0241962.s002.pdf]

# PROSPERO International prospective register of systematic reviews

## Review title and timescale

1 Review title
Give the working title of the review. This must be in English. Ideally it should state succinctly the interventions or exposures being reviewed and the associated health or social problem being addressed in the review.
Pelvic floor muscle exercise in hyperglycemic pregnant women with urinary incontinence. Systematic review

2 Original language title
For reviews in languages other than English, this field should be used to enter the title in the language of the review. This will be displayed together with the English language title.
Portuguese

3 Anticipated or actual start date
Give the date when the systematic review commenced, or is expected to commence.
01/08/2017

4 Anticipated completion date
Give the date by which the review is expected to be completed.
01/08/2018

5 Stage of review at time of this submission
Indicate the stage of progress of the review by ticking the relevant boxes. Reviews that have progressed beyond the point of completing data extraction at the time of initial registration are not eligible for inclusion in PROSPERO. This field should be updated when any amendments are made to a published record.

The review has not yet started √

| Review stage | Started | Completed |
|---|---|---|
| Preliminary searches | No | No |
| Piloting of the study selection process | No | No |
| Formal screening of search results against eligibility criteria | No | No |
| Data extraction | No | No |
| Risk of bias (quality) assessment | No | No |
| Data analysis | No | No |

Provide any other relevant information about the stage of the review here.

## Review team details

6 Named contact
The named contact acts as the guarantor for the accuracy of the information presented in the register record.
Gabriela Andrade Piemonte

7 Named contact email
Enter the electronic mail address of the named contact.
gabriela@unoeste.br

8 Named contact address
Enter the full postal address for the named contact.
Moacir bezerra dos Anjos 235 Presidente Prudente - SP - Brazil Zip Code:19.053-709

9 Named contact phone number
Enter the telephone number for the named contact, including international dialing code.
55 18 981145478

10 Organisational affiliation of the review
Full title of the organisational affiliations for this review, and website address if available. This field may be completed as 'None' if the review is not affiliated to any organisation.

São Paulo State University - UNESP

Website address:
www.fmb.unesp.br

11 Review team members and their organisational affiliations
Give the title, first name and last name of all members of the team working directly on the review. Give the organisational affiliations of each member of the review team.

| Title | First name | Last name | Affiliation |
|---|---|---|---|
| Dr | Gabriela | Andrade Piemonte | Oeste Paulista University - UNOESTE |
| Dr | Bary | Berghmans | Maastricht University |
| Dr | Rob | A de Bie | Maastricht University |
| Dr | Alvaro | Nagib Atallah | Universidade Federal de São Paulo |
| Dr | Marilza | Vieira Cunha Rudge | São Paulo State University |
| Dr | Angélica | Mércia Pascon Barbosa | São Paulo State University |

12 Funding sources/sponsors
Give details of the individuals, organizations, groups or other legal entities who take responsibility for initiating, managing, sponsoring and/or financing the review. Any unique identification numbers assigned to the review by the individuals or bodies listed should be included.
Funding privided by Fundação de Amparo à Pesquisa do Estado de São Paulo - FAPESP 2016/01743-5

13 Conflicts of interest
List any conditions that could lead to actual or perceived undue influence on judgements concerning the main topic investigated in the review.
Are there any actual or potential conflicts of interest?
None known

14 Collaborators
Give the name, affiliation and role of any individuals or organisations who are working on the review but who are not listed as review team members.

| Title | First name | Last name | Organisation details |
|---|---|---|---|
| Dr | | | |

## Review methods

15 Review question(s)
State the question(s) to be addressed / review objectives. Please complete a separate box for each question.
Is exercise of pelvic floor muscles effective and safe for urinary incontinence in hyperglycemic pregnant women?

16 Searches
Give details of the sources to be searched, and any restrictions (e.g. language or publication period). The full search strategy is not required, but may be supplied as a link or attachment.
We will search the following electronic bibliographic databases: MEDLINE, EMBASE, LILACS ( Latino-American and Caribbean Literature in Health Science), The Cochrane Library (Cochrane Database of Systematic Reviews, Cochrane Central Register of Controlled Trials (CENTRAL), Cochrane Methodology Register), CINAHL (Cumulate Index to Nursisg and Allied Health Literature), PEDro (Physiotherapy Evidence Database), Clinical Trials and ICTRP-WHO. The search strategy will include only terms relating to or describing the intervention. The terms will be combined with the Cochrane MEDLINE filter for controlled trials of interventions. The search strategy for MEDLINE is available in the published protocol. The search terms will be adapted for use with other bibliographic databases in combination with database-specific filters for controlled trials, where these are available. There will be no language and dates restrictions. The searches will be re-run just before the final analyses and further studies retrieved for inclusion.

17 URL to search strategy
If you have one, give the link to your search strategy here. Alternatively you can e-mail this to PROSPERO and we

will store and link to it.

I give permission for this file to be made publicly available
Yes

18 Condition or domain being studied
Give a short description of the disease, condition or healthcare domain being studied. This could include health and wellbeing outcomes.
Urinary incontinence. Hyperglycemic pregnant women.

19 Participants/population
Give summary criteria for the participants or populations being studied by the review. The preferred format includes details of both inclusion and exclusion criteria.
Hyperglycemic pregnant women at any gestational age with urinary incontinence.

20 Intervention(s), exposure(s)
Give full and clear descriptions of the nature of the interventions or the exposures to be reviewed
Any kind of pelvic floor muscles exercises at least once a week with ou without supervision.

21 Comparator(s)/control
Where relevant, give details of the alternatives against which the main subject/topic of the review will be compared (e.g. another intervention or a non-exposed control group).
Another intervention or a non-exposed control group.

22 Types of study to be included
Give details of the study designs to be included in the review. If there are no restrictions on the types of study design eligible for inclusion, this should be stated.
We will include randomised trials to assess the beneficial effects of the treatments, and will supplement these with observational studies (including cohort and case–control studies) for the assessment of harms

23 Context
Give summary details of the setting and other relevant characteristics which help define the inclusion or exclusion criteria.

24 Primary outcome(s)
Give the most important outcomes.
Symptom scales, cure and adverses events.

Give information on timing and effect measures, as appropriate.
Pad test, voiding diary, urodynamic study, pacient self report.

25 Secondary outcomes
List any additional outcomes that will be addressed. If there are no secondary outcomes enter None.
pelvic floor muscle strength and quality of life.

Give information on timing and effect measures, as appropriate.
fucntional avaliation, perineometer, eletromiography, functional ultrasonography, and any kind of questionnaire used to avaliate the impacty of the urinary incontinence in quality of life.

26 Data extraction (selection and coding)
Give the procedure for selecting studies for the review and extracting data, including the number of researchers involved and how discrepancies will be resolved. List the data to be extracted.
Titles and/or abstracts of studies retrieved using the search strategy and those from additional sources will be screened independently by two review authors to identify studies that potentially meet the inclusion criteria outlined above. The full text of these potentially eligible studies will be retrieved and independently assessed for eligibility by two review team members. Any disagreement between them over the eligibility of particular studies will be resolved through discussion with a third reviewer.A standardised form will be used to extract data from the included studies for assessment of study quality and evidence synthesis. Extracted information will include: study setting; study population and participant demographics and baseline characteristics; details of the intervention and control conditions; study methodology; recruitment and study completion rates; outcomes and times of measurement; indicators of acceptability to users; suggested mechanisms of intervention action; information for assessment of the

risk of bias. Two review authors will extract data independently, discrepancies will be identified and resolved through discussion (with a third author where necessary). Missing data will be requested from study authors.

27 Risk of bias (quality) assessment

State whether and how risk of bias will be assessed, how the quality of individual studies will be assessed, and whether and how this will influence the planned synthesis.

Two review authors will independently assess the risk of bias in included studies by considering the following characteristics: Randomisation sequence generation; Treatment allocation concealment; Blinding; Completeness of outcome data; Selective outcome reporting; and Other sources of bias. Disagreements between the review authors over the risk of bias in particular studies will be resolved by discussion, with involvement of a third review author where necessary.

28 Strategy for data synthesis

Give the planned general approach to be used, for example whether the data to be used will be aggregate or at the level of individual participants, and whether a quantitative or narrative (descriptive) synthesis is planned. Where appropriate a brief outline of analytic approach should be given.

We will provide a narrative synthesis of the findings from the included studies, structured around the type of intervention, target population characteristics, type of outcome and intervention content. We will provide summaries of intervention effects for each study by calculating risk ratios (for dichotomous outcomes) or standardised mean differences (for continuous outcomes). We anticipate that there will be limited scope for meta-analysis because of the range of different outcomes measured across the small number of existing trials. However, where studies have used the same type of intervention and comparator, with the same outcome measure, we will pool the results using a random-effects meta-analysis, with standardised mean differences for continuous outcomes and risk ratios for binary outcomes, and calculate 95% confidence intervals and two sided P values for each outcome. In studies where the effects of clustering have not been taken into account, we will adjust the standard deviations for the design effect. Heterogeneity between the studies in effect measures will be assessed using both the ?2 test and the I2 statistic. We will consider an I2 value greater than 50% indicative of substantial heterogeneity. We will conduct sensitivity analyses based on study quality. We will use stratified meta-analyses to explore heterogeneity in effect estimates according to: study quality; study populations; the logistics of intervention provision; and intervention content. We will also assess evidence of publication bias.

29 Analysis of subgroups or subsets

Give any planned exploration of subgroups or subsets within the review. 'None planned' is a valid response if no subgroup analyses are planned.

If the necessary data are available, subgroup analyses will be done. This is a qualitative synthesis and while subgroup analyses may be undertaken it is not possible to specify the groups in advance.

## Review general information

30 Type and method of review

Select the type of review and the review method from the drop down list.

Systematic review

Physiotherapy

31 Language

Select the language(s) in which the review is being written and will be made available, from the drop down list. Use the control key to select more than one language.

Portuguese-Brazil

Will a summary/abstract be made available in English?

Yes

32 Country

Select the country in which the review is being carried out from the drop down list. For multi-national collaborations select all the countries involved. Use the control key to select more than one country.

Brazil

33 Other registration details

Give the name of any organisation where the systematic review title or protocol is registered together with any unique

identification number assigned. If extracted data will be stored and made available through a repository such as the Systematic Review Data Repository (SRDR), details and a link should be included here.

34 Reference and/or URL for published protocol
Give the citation for the published protocol, if there is one.
Give the link to the published protocol, if there is one. This may be to an external site or to a protocol deposited with CRD in pdf format.

I give permission for this file to be made publicly available
Yes

35 Dissemination plans
Give brief details of plans for communicating essential messages from the review to the appropriate audiences.
Do you intend to publish the review on completion?
Yes

36 Keywords
Give words or phrases that best describe the review. (One word per box, create a new box for each term)

37 Details of any existing review of the same topic by the same authors
Give details of earlier versions of the systematic review if an update of an existing review is being registered, including full bibliographic reference if possible.

38 Current review status
Review status should be updated when the review is completed and when it is published.
Ongoing

39 Any additional information
Provide any further information the review team consider relevant to the registration of the review.

40 Details of final report/publication(s)
This field should be left empty until details of the completed review are available.
Give the full citation for the final report or publication of the systematic review.
Give the URL where available.
