# Supplementary material for: Effectiveness of the pelvic floor muscle training on muscular dysfunction and pregnancy specific urinary incontinence in pregnant women with gestational diabetes mellitus: A systematic review protocol
Source: PLoS One. 2020 Dec 7;15(12):e0241962. doi: 10.1371/journal.pone.0241962 (PMC7721159; doi:10.1371/journal.pone.0241962)
Supplement: Attachment — Submitted filename: reviewer comment.docx [file pone.0241962.s003.docx]

**2020.07.09**

**Reviewer comment**

**Dear Editor**

Thank you for giving me the opportunity to review this article.

Very good paper. It addresses an issue that is neglected in many societies.

I have a few minor points. Hope these comments will be helpful to the authors.

Please read the following information and revise your manuscript as necessary.

Please explain more about the type of PFM training and the duration of it in detail.

The MeSH terms used will be "Pregnancy", "Hyperglycemia", "Diabetes Mellitus, Type 2", "Diabetes Mellitus, Type 1", "Pregnancy in Diabetics", "Diabetes, Gestational" and "Urinary Incontinence".

The search strategy does not mention the keywords "pelvic floor muscle strength" and "quality of life". I have the impression that it is better to mention them.

The quality score of the articles is not recorded in the item tables of study characteristics. It is better to added.

Who are two independent investigators to check the quality of evidence?
It not clear if all types of urinary incontinence in pregnancy considered or stress urinary incontinence? It is not explicitly mentioned in study inclusion and exclusion criteria.
If yes. Do the authors intend to compare subgroups?

Given that the type of PFM training and the duration of it affects the outcome. How can this be considered? It is better to be explained.

With respect
